# Differential degradation of petroleum hydrocarbons by *Shewanella putrefaciens* under aerobic and anaerobic conditions

**DOI:** 10.3389/fmicb.2024.1389954

**Published:** 2024-04-10

**Authors:** Yang Li, Yuan Liu, Dongyi Guo, Hailiang Dong

**Affiliations:** ^1^Center for Geomicrobiology and Biogeochemistry Research, State Key Laboratory of Biogeology and Environmental Geology, China University of Geosciences, Beijing, China; ^2^School of Earth Sciences and Resources, China University of Geosciences, Beijing, China

**Keywords:** facultative bacteria, saturates, aromatics, aerobic degradation, anaerobic degradation

## Abstract

The complexity of crude oil composition, combined with the fluctuating oxygen level in contaminated environments, poses challenges for the bioremediation of oil pollutants, because of compound-specific microbial degradation of petroleum hydrocarbons under certain conditions. As a result, facultative bacteria capable of breaking down petroleum hydrocarbons under both aerobic and anaerobic conditions are presumably effective, however, this hypothesis has not been directly tested. In the current investigation, *Shewanella putrefaciens* CN32, a facultative anaerobic bacterium, was used to degrade petroleum hydrocarbons aerobically (using O_2_ as an electron acceptor) and anaerobically (using Fe(III) as an electron acceptor). Under aerobic conditions, CN32 degraded more saturates (65.65 ± 0.01%) than aromatics (43.86 ± 0.03%), with the following order of degradation: dibenzofurans > *n*-alkanes > biphenyls > fluorenes > naphthalenes > alkylcyclohexanes > dibenzothiophenes > phenanthrenes. In contrast, under anaerobic conditions, CN32 exhibited a higher degradation of aromatics (53.94 ± 0.02%) than saturates (23.36 ± 0.01%), with the following order of degradation: dibenzofurans > fluorenes > biphenyls > naphthalenes > dibenzothiophenes > phenanthrenes > *n*-alkanes > alkylcyclohexanes. The upregulation of 4-hydroxy-3-polyprenylbenzoate decarboxylase (ubiD), which plays a crucial role in breaking down resistant aromatic compounds, was correlated with the anaerobic degradation of aromatics. At the molecular level, CN32 exhibited a higher efficiency in degrading *n*-alkanes with low and high carbon numbers relative to those with medium carbon chain lengths. In addition, the degradation of polycyclic aromatic hydrocarbons (PAHs) under both aerobic and anaerobic conditions became increasingly difficult with increased numbers of benzene rings and methyl groups. This study offers a potential solution for the development of targeted remediation of pollutants under oscillating redox conditions.

## Highlights

- CN32 has the ability to degrade crude oil using both O_2_ and Fe(III) as electron acceptors.
- CN32 promoted the breakdown of saturates under aerobic conditions.
- CN32 degraded aromatics more efficiently under anaerobic conditions.

## 1 Introduction

Petroleum hydrocarbons are complex mixtures consisting predominantly of saturates and aromatics (Strausz and Lown, 2003; Mbadinga et al., 2011; Liu et al., 2024). Over the last few decades, as the demand for petroleum products continues to rise, global oil production has surpassed 5 billion tons per year (BP, 2022). Every year, approximately 8 million tons of petroleum hydrocarbons pollute water and land as a result of exploration, production, storage, and transportation (Varjani, 2016). Petroleum compounds can be teratogenic, carcinogenic, and mutagenic, thus posing harm to both the ecological environment and human health (Khudur et al., 2019). For instance, 16 unbranched polycyclic aromatic hydrocarbons (PAHs) are listed as priority pollutants by the Environmental Protection Agency (EPA) of the United States (ATSDR, 2005). Bioremediation has emerged as a technology for removing petroleum pollutants from contaminated water, sediments, and soils because of its simplicity, cost-effectiveness, and environmental friendliness (Varjani, 2016; Khudur et al., 2019). Bacteria are more frequently and extensively employed as degraders compared to algae, fungi, and archaea (Brooijmans et al., 2009; Das and Chandran, 2011), because they can use petroleum hydrocarbons as the sole carbon and energy source (Mbadinga et al., 2011) when coupled with oxygen (Setti et al., 1993; Díaz et al., 2013), sulfate (Aeckersberg et al., 1991; Rueter et al., 1994), nitrate (Grishchenkov et al., 2000; Coates et al., 2001), and iron (Lovley et al., 1989; Anderson and Lovley, 1999; Liu et al., 2023a) as electron acceptors.

Compared with sulfate and nitrate, iron is more abundant in the crust [35% of the total mass of the Earth (Coey, 1980)], because they are present as both dissolved iron and solid forms in iron-bearing clay minerals and iron oxides in oxidized (Fe(III)) or reduced (Fe(II)) states (Davison, 1993; Weber et al., 2006; Dong et al., 2022). Bacteria play important roles in cycling iron between Fe(III) and Fe(II) (Dong et al., 2023b). For example, Fe(III)-reducing bacteria are found in oil-contaminated environments because of the presence of abundant oil compounds as electron donors (Martín-Gil et al., 2004; Kleemann and Meckenstock, 2011). Hence, Fe(III) can serve as the potential electron acceptor to couple with petroleum biodegradation (Lovley et al., 1989; Vodyanitskii, 2011; Liu et al., 2023a). Indeed, oil-contaminated sites often contain dissolved iron, with concentration levels ranging from 0.1 mM (Qian et al., 2018) to as high as 1 mM (Bennett et al., 1993). These high levels of dissolved iron may be a result of transformation of iron-bearing minerals through two mechanisms: (1) organic acids produced from microbial metabolism facilitate dissolution of iron-bearing minerals (Barth and Riis, 1992); (2) bio-reduction of Fe(III)-bearing minerals leads to reductive dissolution of iron-bearing minerals (Weber et al., 2006; Zeng et al., 2016, 2020). Furthermore, it may be feasible to amend dissolved iron for the purpose of degrading crude oil contamination (Yan et al., 2017; Zhang et al., 2021). However, the role of dissolved Fe(III) in oil degradation has not been fully studied, despite its higher bioavailability compared to iron-bearing minerals (Takahashi et al., 2011).

Oil-contaminated field sites encompass various environments with a range of oxygen levels, including aerobic surficial soil (Crowe and Smith, 2007), transition zones between aerobic and anaerobic settings such as water-bearing soil (Tong et al., 2016; Sheng et al., 2021b), and anaerobic habitats such as deep sea (Ma et al., 2021) and groundwater (Lovley and Lonergan, 1990; Sheng et al., 2016). Moreover, even in the same location, oxygen fugacity can change, leading to transition between aerobic and anaerobic conditions (Davison, 1993; Meckenstock and Mouttaki, 2011). However, previous studies have focused on biodegradation of oil compounds by the obligate bacteria under either aerobic or anaerobic conditions (Haritash and Kaushik, 2009; Xu et al., 2018), which prevents their applicability to oil-polluted environments with fluctuating oxygen conditions.

Facultative anaerobic bacteria exhibit higher adaptability to environmental changes compared to obligate bacteria (Cason et al., 2019). Several studies have shown that facultative anaerobes have the capability to biodegrade specific PAH compounds, such as phenanthrene, pyrene, and benzo[*a*]pyrene (BaP), under both aerobic and Fe(III) reduction conditions (Liang et al., 2014; Yan et al., 2017; Zhang et al., 2021). However, crude oil is a complex mixture of many different compounds with a wide range of solubilities. It has been challenging to find a bacterium capable of degrading a range of petroleum compounds under oscillating redox conditions. *Shewanella putrefaciens*, a representative typical facultative anaerobe, has demonstrated its capability to separately degrade dibenzothiophenes under aerobic conditions (Ansari et al., 2007) and petroleum hydrocarbons under anaerobic conditions (Liu et al., 2023a). Hence, *Shewanella putrefaciens* has the potential to degrade complex organic compounds of crude oil in both aerobic and anaerobic environments. However, its ability to degrade the same petroleum hydrocarbons under both aerobic and anaerobic conditions has not been demonstrated.

This study aimed to evaluate the biodegradation potential of complex oil compounds by facultative anaerobes using either O_2_ or Fe(III) as the electron acceptor, employing *Shewanella putrefaciens* CN32 isolated from an anaerobic core sample 250 feet below the Morrison Formation in northwestern New Mexico (Zachara et al., 1998) as a model organism. Gas chromatography-mass spectrometer (GC–MS) was used to monitor changes in the concentration of oil compounds under two conditions. To unravel the biodegradation mechanism, quantitative reverse transcription polymerase chain reaction (RT-qPCR) was employed to examine the differential expression of specific genes responsible for biodegradation under both conditions. The findings of this study provide theoretical support for bioremediation of oil contamination. Moreover, this study sheds light on the significant role of facultative anaerobes in biogeochemical carbon and iron cycles under oxygen oscillating conditions.

## 2 Materials and methods

### 2.1 Preparation of crude oil, CN32 cells, and ferric citrate stock solution

A crude oil sample collected from the Changqing oilfield served as the substrate for the biodegradation experiments under aerobic and anaerobic conditions. CN32 cells were aerobically cultured in Luria-Bertani (LB) broth at pH 7.0. The culture was incubated at 30°C and agitated at 150 rpm in a constant temperature incubator (HZQ-X100, Suzhou Peiying Experimental Equipment Co., LTD, Suzhou, China). After reaching the logarithmic phase (approximately 16 h of growth), CN32 cells were centrifuged at 5,000 g for 5 min. The cells were then re-suspended in sterile bicarbonate buffer (consisting of 30 mM NaHCO_3_, 10 mM KCl, pH = 7.0) to remove residual LB medium. The centrifugation-resuspension process was repeated three times under either aerobic or anaerobic condition and the final cell pellet was re-suspended in the same buffer for the subsequent experiments. The aerobic experiment was conducted on a clean workbench (*VS*-1300 L-U, Suzhou Antai Air Technology Co., LTD, Suzhou, China). The anaerobic experiment was carried out inside an anaerobic chamber (filled with 98% N_2_ and 2% H_2_, Coy Laboratory Products, Grass Lake, Michigan). To prepare anoxic soluble Fe(III) stock solution, ferric citrate (>99%, Sigma) was dissolved in ddH_2_O (18 MΩ cm) to achieve 100 mM concentration. The solution was sterilized by passing it through a 0.22 μm filter.

### 2.2 Biodegradation experiments

#### 2.2.1 Aerobic experiment

Conical flasks (250 mL) were prepared, and 2 g of crude oil and 90 mL of basic medium (pH = 7.0) were added to the flasks. The concentration of crude oil, at 20 grams per liter, was much higher than those used in most studies (Wang et al., 2020; Liu et al., 2020a, 2023a). This higher concentration was chosen to represent a heavily polluted environment. The basic medium consisted of the following components: 3 g/L KNO_3_, 2 g/L Na_2_HPO_4_, 2 g/L KH_2_PO_4_, 0.5 g/L MgSO_4_, 0.5 g/L NaCl, 1 g/L NH_4_Cl, and a 10 mL trace elements solution (Liu et al., 2020b). The trace element solution included the following components: 12 g/L Na_2_EDTA·2H_2_O, 2 g/L NaOH, 1 g/L CaCl_2_, 0.4 g/L MnSO_4_·4H_2_O, 0.4 g/L ZnSO_4_·7H2O, 0.5 mL H_2_SO_4_ (98%), 10 g/L Na_2_SO_4_, 0.1 g/L Na_2_MoO_4_·2H_2_O, 2 g/L FeSO_4_·7H_2_O, and 0.1 g/L CuSO_4_·5H_2_O (Liu et al., 2020b).

The prepared flasks containing the mixture of the crude oil and the basic medium were autoclaved (121°C for 30 min) (autoclave model: PHCbi MLLS-3718 L). After adding 10 mL cells (final concentration 10^8^ CFU/mL), the flasks were aerobically incubated for 30 days at a temperature of 30°C and a speed of 150 rpm in an HZQ-X100 incubator. On day 16, new CN32 cells were introduced into flasks to maintain an adequate viable cell concentration. To establish an abiotic control, CN32 cells were replaced with sterilized distilled water (ddH_2_O). All experiments were conducted in triplicates.

#### 2.2.2 Anaerobic experiment

In the experimental setup, 150 mL serum bottles were used for the anaerobic degradation experiment. The serum bottles contained 20 g/L crude oil and 85 mL basic medium with a pH 6.9. The basic medium consisted of the following components: NaHCO_3_ (2.5 g/L), CaCl_2_·2H_2_O (0.09 g/L), NH_4_Cl (1.0 g/L), MgCl_2_·6H_2_O (0.2 g/L), NaCl (10 g/L), HEPES (7.2 g/L), K_2_HPO_4_ (36 mg/L), Na_2_SO_4_ (0.71 mg/L), and a trace mineral solution (10 mL). The trace mineral solution contained FeCl_2_·4H_2_O (0.2 g/L), Na_2_WO_4_·2H_2_O (0.02 g/L), MnCl_2_·4H_2_O (0.1 g/L), CoCl_2_·6H_2_O (0.1 g/L), ZnCl_2_ (0.05 g/L), CuCl_2_·2H_2_O (0.002 g/L), H_3_BO_3_ (0.005 g/L), Na_2_MoO_4_·2H_2_O (0.01 g/L), Na_2_SeO_3_ (0.017 g/L), NiCl_2_·6H_2_O (0.024 g/L), and nitrilotriacetic acid (1.5 g/L) (Saito et al., 2016).

To create an anaerobic environment, the serum bottles were purged with anoxic N_2_ to remove oxygen. After purging, the bottles were tightly sealed using a butyl rubber septum and an aluminum crimp. To ensure sterility, the sealed bottles were then autoclaved at a temperature of 121°C for 30 min. The serum bottles were prepared by combining 10 mL washed cells (at a final cell concentration approximately 10^8^ CFU/mL) with 5 mL of ferric citrate solution (at a final concentration of 5 mM). The serum bottles were anaerobically incubated in the dark at 30°C and 150 rpm in an HZQ-X100 incubator for 30 days. On day 16, new CN32 cells were introduced into the serum bottles to maintain an adequate viable cell concentration. To establish an abiotic control, anoxic sterilized ddH_2_O was used as a substitute for CN32 cells. All experiments were conducted in triplicates.

### 2.3 Analytical methods

#### 2.3.1 Total Fe(II) and total Fe concentrations

Fe(II) concentration was measured to monitor Fe(III) reduction. Periodically, sub-samples (0.2 mL) were aseptically withdrawn from the serum bottles using sterile needles and syringes in an anaerobic chamber. The collected samples were then analyzed using the 1,10-phenanthroline method to determine the concentrations of total Fe(II) and total Fe (Amonette and Templeton, 1998). To determine the total Fe(II) content, a 0.2 mL of suspension sample was combined with 0.48 mL H_2_SO_4_ (3.6 N) in an Eppendorf tube. This mixture was then supplemented with 40 μL HF (40%, w/v) and 80 μL 1,10-phenanthroline reagent (10%, w/v). The tubes were then exposed to boiling at 100°C for 30 min and allowed to cool for 15 min at room temperature. Following these steps, 0.4 mL H_2_BO_3_ (5%, w/v) was added. Subsequently, 0.1 mL of the mixture was transferred to a separate tube containing 1 mL Na-citrate (1%, w/v). After reaction for 20 min, the total Fe(II) content was measured using a UV–Vis spectrophotometer (SHIMADZU2550, Japan) at 510 nm wavelength. To determine the total Fe concentration [Fe(III) and Fe(II)], the Fe(III) was first reduced to Fe(II) by adding hydroxylamine hydrochloride (10% in 1% Na-citrate, w/v). The extent of Fe(III) reduction was calculated using the following equation:


$$Fe\left(III\right)\;\text{reduction}\;\text{extent}\;\left(\%\right)=\frac{\left(Fe{\left(II\right)}_{\text{total}}-Fe{\left(II\right)}_{\text{initial}}\right)}{{Fe}_{\text{total}}}*100\%$$

#### 2.3.2 Changes in petroleum hydrocarbons induced by O_2_ and Fe(III) reduction

GC–MS was used to identify and quantify the changes in the composition of saturates and aromatics. The biodegraded oil samples that underwent 30-day degradation under aerobic and anaerobic conditions were labeled as A-30 and An-30, respectively. Following the 30-day biodegradation period, 30 mL dichloromethane was added to 10 mL suspension to extract the crude oil. A detailed extraction method was described previously (Liu et al., 2020b). The extracted oil was fractionated by column chromatography to collect saturates, aromatics, resins, and asphaltenes (Liu et al., 2020b). Saturates and aromatics from both A-30 and An-30 were analyzed using a GC–MS system (Agilent 6890/5975) (Supplementary material Text S1; Liu et al., 2020b). To enable semi-quantification of the saturated and aromatic fractions, deuterium *n*C_24_ (CAS: 16416–32-3; Chiron) and deuterium dibenzothiophene (CAS: 33262–29-2; Chiron) were used, respectively. For the *m*/*z* and abbreviations of the representative compound series, please refer to Liu et al. (2023a). The concentration (μg/g oil) and biodegradation ratio (%) of specific hydrocarbon compounds were calculated using the following equations outlined in Liu et al. (2023a):


$$\frac{C_{X}}{C_{I}}=k*\frac{S_{X}}{S_{I}}\;\left(C_{I}=\frac{m_{I}}{m_{0}}\right)\text{;}$$

$$\text{Biodegradation}\;\text{ratio}\;\left(\%\right)=\frac{C_{0}-C_{X}}{C_{0}}*100\%$$

where *C_X_* and *C_I_* are the concentrations (μg/g) of the compound in crude oil and the internal standard, respectively; *S_X_* and *S_I_* are the peak areas of each compound in crude oil and the internal standard, respectively; *k* is the response factor (assumed to be 1.0); *m_I_* and *m*_0_ represent the masses of the internal standard and oil samples, respectively; *C*_0_ and *C_X_* are the concentrations of the compounds in fresh and biodegraded crude oils (μg/g), respectively.

#### 2.3.3 Quantitative reverse transcription polymerase chain reaction (RT-qPCT)

The 4-hydroxy-3-polyprenylbenzoate decarboxylase (ubiD) is present in the genome of the strain CN32 (Data from the NCBI database).^1^ It is responsible for anaerobic carboxylation of benzene, naphthalene, and phenanthrene (Atashgahi et al., 2018; Koelschbach et al., 2019; Zhang et al., 2021), and it is also expressed in aerobic ubiquinone biosynthesis pathway in *Escherichia coli* (Arias-Cartin et al., 2023). Since the CN32 strain is facultative anaerobe, ubiD gene might play an unknown role for aromatic degradation under aerobic conditions. Therefore, ubiD was used to evaluate the degradation characteristics of aromatics under different conditions. RT-qPCR was employed to quantify the expression of the ubiD gene under aerobic and anaerobic conditions.

Specifically, total RNA of strain CN32 was extracted using UNIQ-10 column Trizol total RNA extraction kit (B511321, Shanghai Sangon Biological Engineering Technology & Services, Co., Ltd., China). The purity (OD_260_/OD_280_) and concentration (ng/μL) of the extracted RNA were determined by a microvolume UV–Vis spectrophotometer (SMA4000, Merinton Instrument Co., Ltd., Beijing, China) (Bezerra et al., 2019). The integrity of RNA was verified by detecting the 16S and 23S bands of rRNA in 1.5% (w/v) agarose gel stained with a nucleic acid dye (Bezerra et al., 2019).

Subsequently, RNA (~500 ng) was reverse-transcribed to cDNA using Random Primer p (dN)_6_ (100 pmol) and Maxima Reverse Transcriptase (200 U; EP0743, Thermo Scientific, Wilmington, DE, United States). Primers of DNA amplification used for this study were designed in Primer Premier 5.0 based on a gene sequence obtained from the National Center for Biotechnology Information (NCBI) GenBank database. The primer sequences for the 16S rRNA gene were as follows: Forward - 5’-TTCAGTAGGGAGGAAAGGGTAA-3′ and Reverse - 5’-CCAGGGCTTTCACATCTCG-3′. For the ubiD gene, the primer sequences were: Forward - 5’-CGCCTGAGGGTTGTTCGT-3′ and Reverse - 5’-GCGGCAGTTCACATCTTCAT-3′. The primers were validated by q-PCR reactions using cDNA as the template.

qPCR reactions were carried out on a QuantStudio™1 Plus qPCR Detection System (Thermo Scientific, Wilmington, DE, USA). SYBR Green qPCR Master Mix (High Rox, B639273, ABI, USA) was used. The q-PCR program consisted of an initial denaturation step at 95°C for 3 min, followed by 45 cycles of amplification with denaturation at 95°C for 15 s and annealing/extension at 60°C for 30 s. Three biological replicates and three technical replicates were performed.

The 16S rRNA gene of the bacterial strain was utilized as a reference to normalize the expression of the target gene, ubiD. In brief, the threshold cycle (Ct) values for both the target (ubiD) and reference genes (16S rRNA) were obtained via qPCR across different samples. The expression of the target gene was then adjusted based on the Ct value of the reference gene (Sheng et al., 2023). The fold change in gene expression was determined using the 2^-ΔΔCt^ method, a well-established technique for relative quantification (Livak and Schmittgen, 2001; Vaishnav et al., 2022). Here, ΔCt refers to the Ct difference between the target gene (ubiD) and the reference gene (16S rRNA gene), while ΔΔCt calculates the ΔCt variation between the anaerobic and aerobic experimental groups.

## 3 Results

### 3.1 Overall degradation characteristics of petroleum hydrocarbons

**Figure 1:**
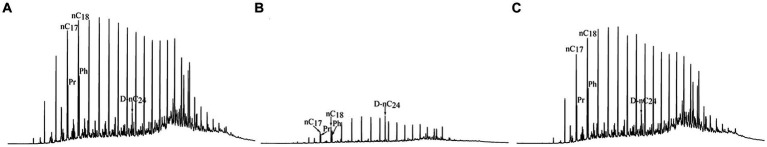
The total ion chromatograms (TICs) of saturates from control oil **(A)**, biodegraded oil after 30 days under aerobic condition **(B)**, and anaerobic condition **(C)**. Pr: pristane, Ph: phytane. Deuterium *n*C_24_ (D-*n*C_24_) is used as an internal standard.

The total ion chromatogram (TIC) of saturated hydrocarbons clearly illustrates the degradation of crude oil under both aerobic and anaerobic conditions (Figure 1). Notably, the TIC reveals a more pronounced degradation under aerobic condition than under anaerobic condition, based on a comparison of the peak height of the internal standard relative to all petroleum hydrocarbon compounds (Figure 1). Because pristane (Pr) and phytane (Ph) have been demonstrated to exhibit bio-resistance (Cabrerizo et al., 2016; Meng et al., 2021), the changes of *n*C_17_/Pr and *n*C_18_/Ph ratios should indicate biodegradation (Kennicutt II et al., 1987; Iheonye et al., 2019). Under aerobic condition, the *n*C_17_/Pr and *n*C_18_/Ph ratios decreased significantly from 2.73 ± 0.07 and 2.50 ± 0.05 to 0.60 ± 0.10 and 0.61 ± 0.10, respectively. However, under anaerobic condition, the *n*C_17_/Pr and *n*C_18_/Ph ratios only decreased to 2.58 ± 0.05 and 2.34 ± 0.04, respectively.

**Figure 2:**
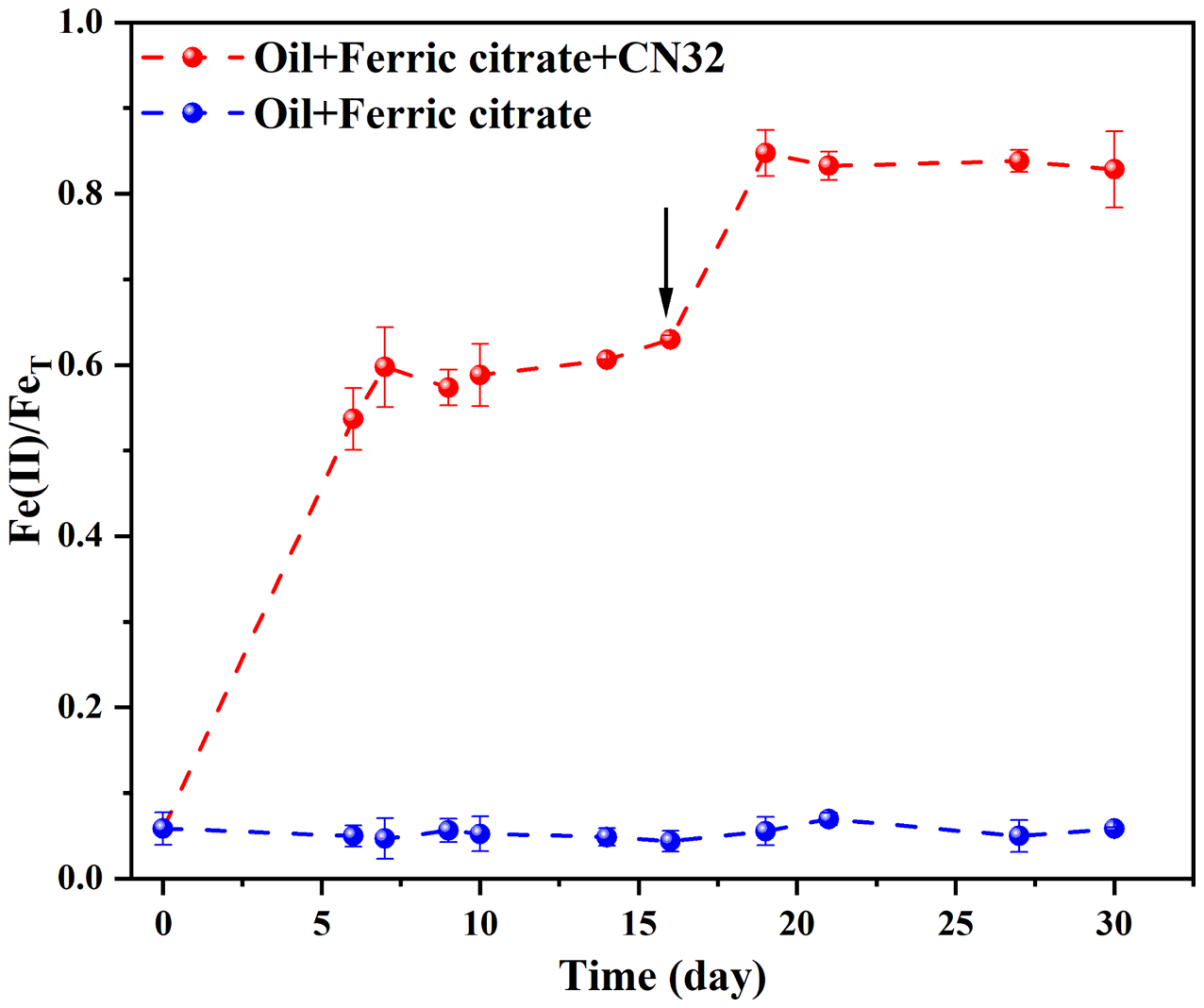
Time-course changes of Fe(II)/Fe_T_ by CN32 under anaerobic condition. The arrow denotes the time of injection of fresh CN32 cells (16 days).

Under anaerobic conditions, the reduction of Fe(III) was coupled with biodegradation of petroleum compounds, reaching a reduction extent of 59.75 ± 0.05% by day 7 (Figure 2). The introduction of fresh CN32 cells on Day 16 led to a subsequent increase in the extent of bio-reduction to 82.82 ± 0.04% by day 30 (Figure 2).

**Figure 3:**
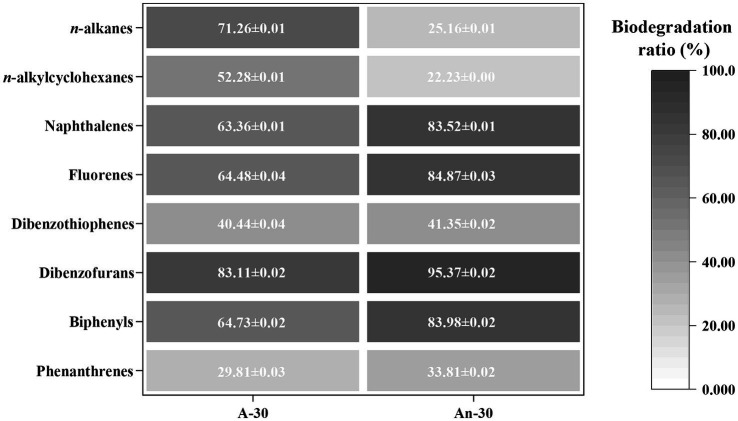
Biodegradation ratios (%) of eight classes of compounds in crude oil by CN32 under aerobic and anaerobic conditions. The saturated hydrocarbons include *n*-alkanes and *n*-alkylcyclohexanes. The aromatic hydrocarbons include two-ring PAHs (naphthalenes, fluorenes, dibenzothiophenes, dibenzofurans, and biphenyls) and three-ring PAHs (phenanthrenes).

The GC–MS semi-quantitative data were employed to characterize changes of concentrations (μg/g) and biodegradation ratios (%) of saturates (i.e., *n*-alkanes and alkylcyclohexane) and aromatics (i.e., PAHs with two and three benzene rings). Specifically, CN32 exhibited differential degradation of eight series of saturated and aromatic hydrocarbons under different conditions. Under aerobic condition, the degradation sequence followed the following order, based on the biodegradation ratio (in parentheses): dibenzofurans (83.11 ± 0.02%) > *n*-alkanes (71.26 ± 0.01%) > biphenyls (64.73 ± 0.02%) > fluorenes (64.48 ± 0.04%) > naphthalenes (63.36 ± 0.01%)  > alkylcyclohexanes (52.28 ± 0.01%) > dibenzothiophenes (40.44 ± 0.04%) > phenanthrenes (33.15 ± 0.04%) (Figure 3). Under anaerobic condition, the degradation sequence followed a different order: dibenzofurans (95.37 ± 0.02%) > fluorenes (84.87 ± 0.03%) > biphenyls (83.98 ± 0.02%) > naphthalenes (83.52 ± 0.01%) > dibenzothiophenes (41.35 ± 0.02%) > phenanthrenes (39.06 ± 0.02%) > *n*-alkanes (25.16 ± 0.01%) > alkylcyclohexanes (22.23 ± 0.00%) (Figure 3). These biodegradation patterns clearly indicate that saturates were more susceptible to degradation under aerobic condition than under anaerobic condition. In contrast, aromatics were more susceptible to degradation under anaerobic condition.

### 3.2 Degradation characteristics of saturated hydrocarbons

**Figure 4:**
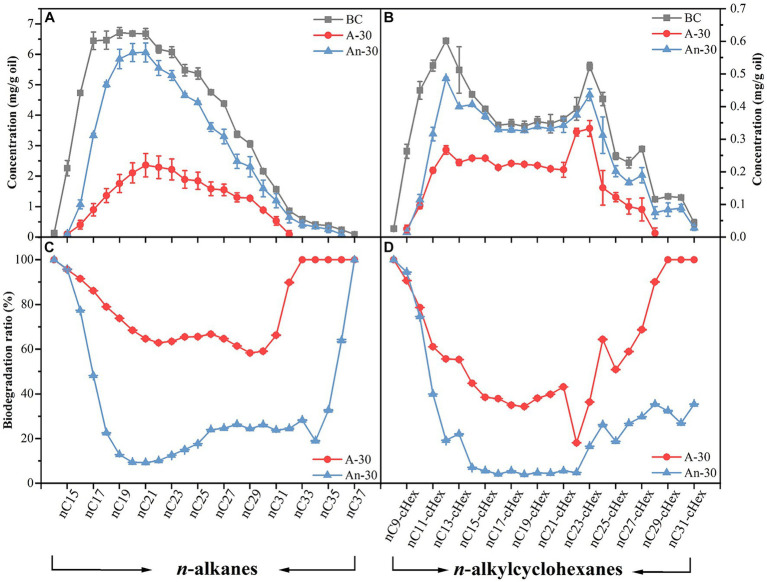
Concentration (mg/g) changes of saturated hydrocarbons due to aerobic (A-30) and anaerobic (An-30) biodegradation by CN32 for 30 days compared to the control oil (BC), including *n*-alkanes **(A)** (left axis) and *n*-alkylcyclohexanes **(B)** (right axis). Biodegradation ratios (%) of *n*-alkanes **(C)** and *n*-alkylcyclohexanes **(D)** under aerobic and anaerobic conditions.

Saturates, mainly comprised of *n*-alkanes and alkylcyclohexanes (del Rio et al., 1994), were more effectively degraded under aerobic condition than under anaerobic condition (Figures 4A,B), consistent with the well-established understanding that aerobic degradation tends to be more efficient for degrading *n*-alkanes (Grishchenkov et al., 2000). The biodegradation ratios of alkylcyclohexanes (aerobic 52.28 ± 0.01%; anaerobic 22.23 ± 0.00%) were lower than those of *n*-alkanes (aerobic 71.26 ± 0.01%; anaerobic 25.16 ± 0.01%) (Figure 3). This result aligns with previous findings that alkylcyclohexanes are more resistant to biodegradation than *n*-alkanes (Kaplan et al., 1997). Some biomarkers, i.e., steranes and hopanes in saturated hydrocarbons (Fu and Sheng, 1989) were not degraded by CN32 (Figure S1 in Supplementary material). Under aerobic condition, the total content of *n*-alkanes decreased significantly from 85.02 mg/g in the abiotic control to 24.43 mg/g in the biotic treatment, representing a total degradation ratio of 71.26 ± 0.01% (Figure 3). Similarly, the total content of alkylcyclohexanes decreased from 7.79 mg/g in the abiotic control to 3.72 mg/g in the biotic treatment, indicating a total degradation ratio of 52.28 ± 0.01% (Figure 3).

At the molecular level, CN32 cells also exhibited higher biodegradation ratios of *n*C_14_-*n*C_37_ compounds under aerobic condition than anaerobic condition (Figure 4C). Nevertheless, both conditions exhibited the following similar degradation patterns. First, *n*C_14_, *n*C_15_, and *n*C_37_ showed degradation ratios close to 100% (Figure 4C). Second, the biodegradation ratios of long- and short-chain *n*-alkanes were consistently higher compared to the *n*-alkanes of intermediate carbon chain length (Figures 4A,C). This trend was also observed in the degradation of *n*C_8_-cHex-*n*C_31_-cHex (Figures 4B,D).

### 3.3 Degradation characteristics of aromatic hydrocarbons

**Figure 5:**
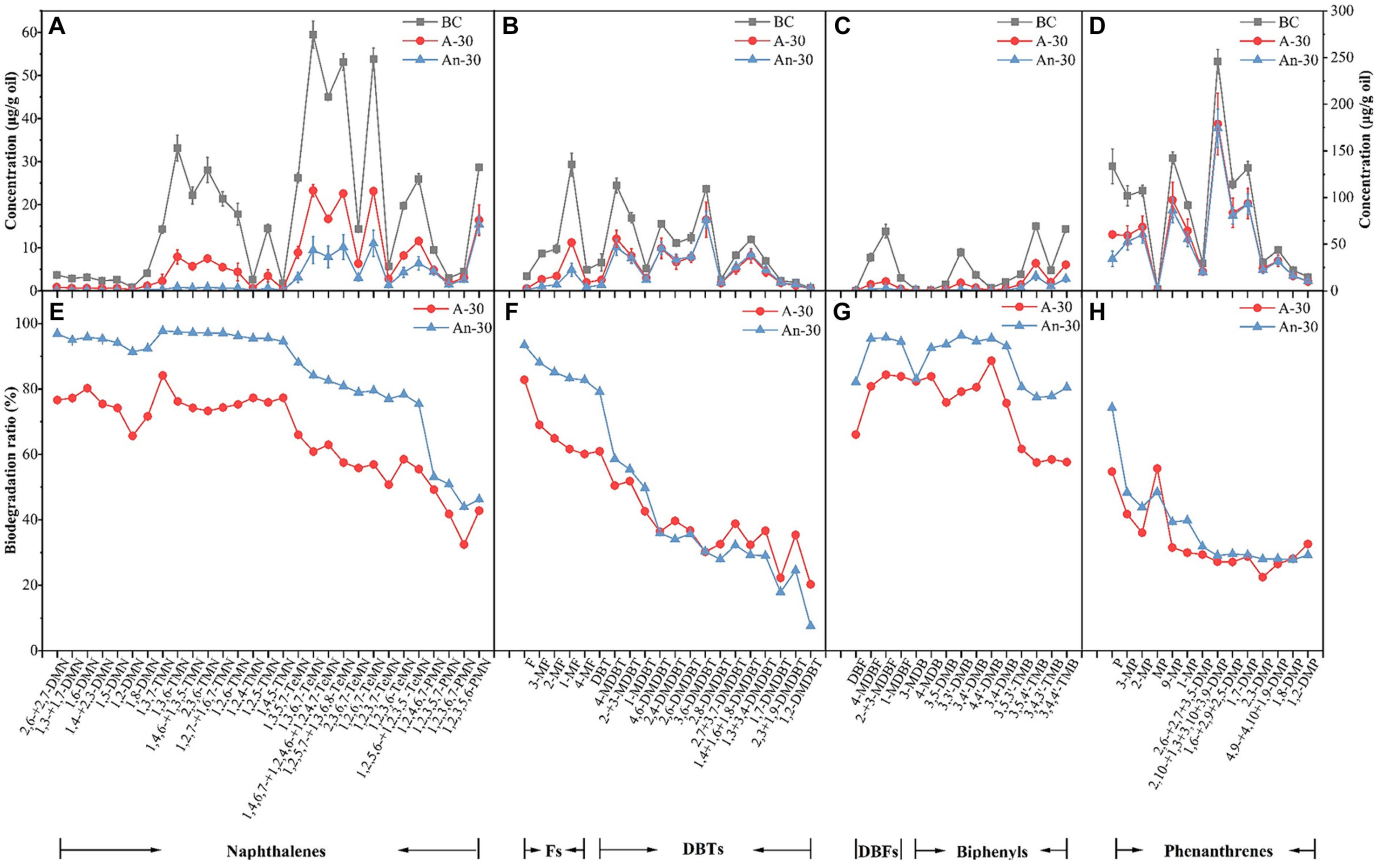
Concentration (μg/g) changes of aromatic hydrocarbons due to aerobic (A-30) and anaerobic (An-30) biodegradation by CN32 after 30 days relative to the control oil (BC), including naphthalenes **(A)**, fluorenes and dibenzothiophenes **(B)**, dibenzofurans and biphenyls **(C)**, and phenanthrenes **(D)**. Biodegradation ratios (%) of aromatic compounds under aerobic and anaerobic conditions **(E–H)**. **(A–C)** refers to the left axis, and (d) refers to the right axis.

**Figure 6:**
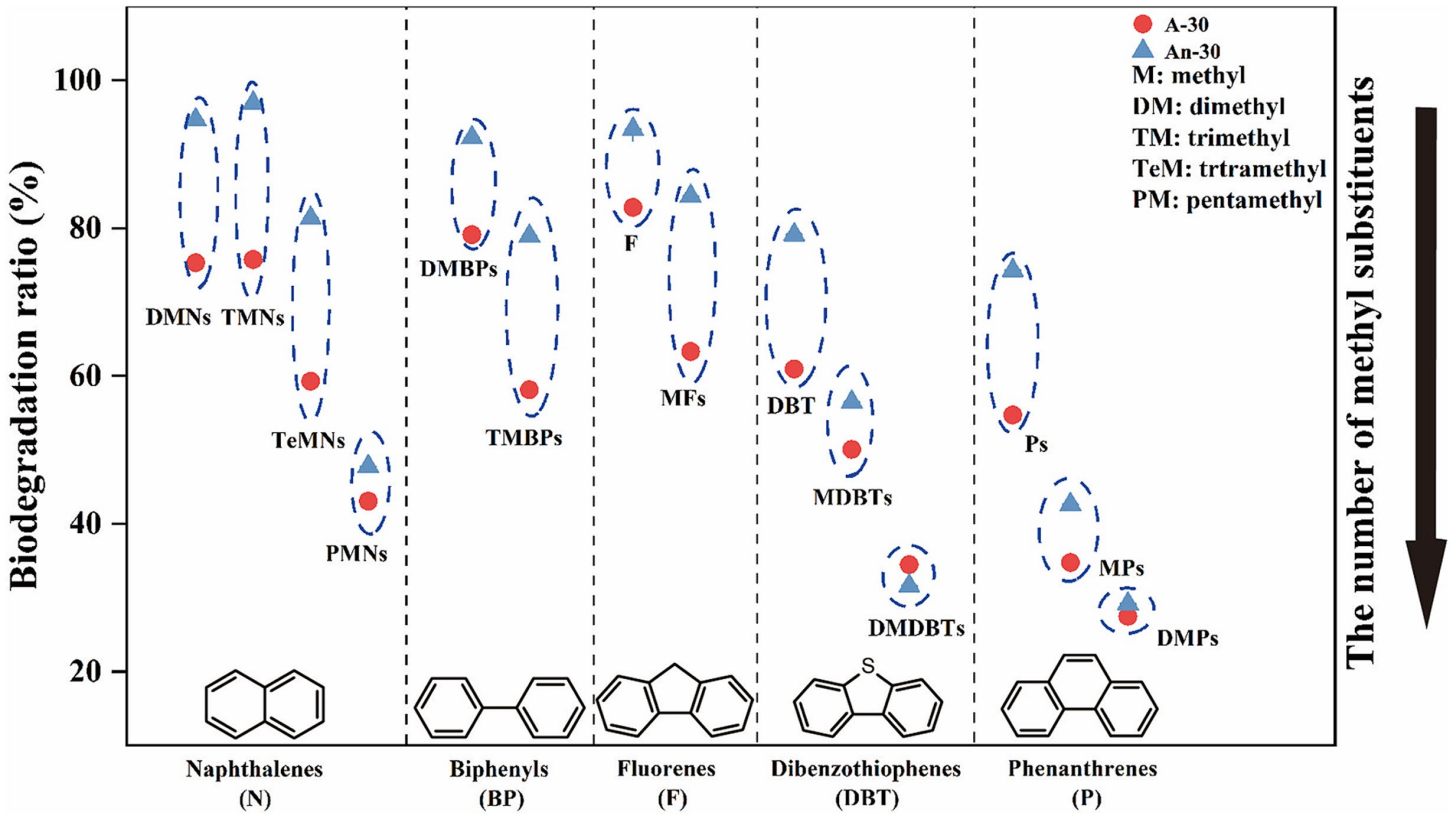
The relationship between the biodegradation ratio and the number of benzene rings and methyl substitutions in PAHs. The biodegradation order of compounds with different number of substitutions is as follow: DMNs, TMNs > TeMNs > PMNs; DMBPs > TMBPs; F > MFs; DBT > MDBTs > DMDBTs; P > MPs > DMPs.

In contrast to the saturates, aromatic hydrocarbons present a significantly higher degradation efficiency by CN32 under anaerobic condition (53.94 ± 0.02%) compared to that in aerobic condition (43.86 ± 0.03%) (Table 1). The studied aromatics with two or three benzene rings showed clear degradation (Figure 3). In general, the concentration (μg/g) and biodegradation ratio (%) of PAHs decreased with the increasing number of benzene rings (Figures 3, 5, 6). Under aerobic condition, three-ring PAHs, such as phenanthrene, exhibited lower degradation ratios (33.15 ± 0.04%) than two-ring PAHs, including dibenzofurans (83.11 ± 0.02%), biphenyls (64.73 ± 0.02%), fluorenes (64.48 ± 0.04%), naphthalenes (63.36 ± 0.01%), and dibenzothiophenes (40.44 ± 0.04%) (Figure 3). Similarly, under anaerobic condition, three-ring PAHs showed lower degradation ratios (phenanthrene: 39.06 ± 0.02%) than two-ring PAHs (dibenzofurans: 95.37 ± 0.02%, fluorenes: 84.87 ± 0.03%, biphenyls: 83.98 ± 0.02%, naphthalenes: 83.52 ± 0.01%, and dibenzothiophenes: 41.35 ± 0.02%) (Figure 3). PAHs with more than three rings were not degraded under both aerobic and anaerobic conditions (Figure S2 in Supplementary material).

**Table 1:** The concentrations and biodegradation ratios of saturates (*n*-alkanes and *n*-alkylcyclohexanes) and aromatics (with two and three benzene rings) by *S. putrefaciens* CN32 under aerobic and anaerobic conditions.

Compound	Oil		A-30				An-30			
	Concentration (mg/g)	Stdev	Concentration (mg/g)	Stdev	Biodegradation ratio (%)	Stdev	Concentration (mg/g)	Stdev	Biodegradation ratio (%)	Stdev
Saturates	99.12	0.70	34.05	1.05	65.65	0.01	76.00	0.94	23.36	0.01
PAHs	2.02	0.03	1.14	0.05	43.86	0.03	0.93	0.04	53.94	0.02

Naphthalenes, a common type of PAHs, consist of various compounds such as dimethylnaphthalenes (DMNs), trimethylnaphthalenes (TMNs), tetramethylnaphthalenes (TeMNs), and pentamethylnaphthalenes (PMNs). While naphthalenes are generally considered to be less toxic compared to other PAHs, their high water solubility and volatility make them more available to receptors (Edwards, 1983). In contrast to saturated hydrocarbons, CN32 demonstrated a higher biodegradation ratio for naphthalenes (83.52 ± 0.01%) under anaerobic condition than under aerobic condition (63.36 ± 0.01%) (Figure 3). Furthermore, naphthalenes with more methyl groups were more resistant to degradation under both conditions (Figures 5A,E, 6). For example, the aerobic biodegradation ratio followed the order: DMNs, TMNs (75.31 ± 0.05% - 75.75 ± 0.02%) > TeMNs (59.28 ± 0.00%) > PMNs (43.05 ± 0.08%) (Figure 6). Similarly, the anaerobic biodegradation ratio followed the order: DMNs, TMNs (94.66 ± 0.01% - 96.91 ± 0.01%) > TeMNs (81.35 ± 0.02%) > PMNs (47.74 ± 0.05%) (Figure 6).

Methyl substitutions occur at its α(1, 4, 5, and 8) and β positions (2, 3, 6, and 7) of naphthalene structures (Liu et al., 2023b). The degradation ratio of alkylnaphthalenes is significantly influenced by the number of methyl substitutions in the ortho position. For instance, TeMNs with a single ortho substitution exhibited higher biodegradation ratios (aerobic: 57.49 ± 0.01% - 60.87 ± 0.01%; anaerobic: 80.87 ± 0.05% - 84.09 ± 0.06%) compared to those with two ortho substitutions (aerobic: 55.48 ± 0.01% - 58.52 ± 0.02%; anaerobic: 75.43 ± 0.02% - 79.48 ± 0.05%) (Figure 5E). The biodegradation efficiencies of PMNs with two ortho substitutions (aerobic: 41.76 ± 0.10% - 49.21 ± 0.09%; anaerobic: 50.84 ± 0.08% - 53.12 ± 0.07%) were higher than those of PMNs with three ortho substitutions (aerobic: 32.45 ± 0.15% - 42.79 ± 0.12%; anaerobic: 43.89 ± 0.07% - 46.23 ± 0.07%) (Figure 5E). Furthermore, the positions of methyl substitutions (i.e., α- or β- positions) influenced the biodegradation ratio. The biodegradation ratio of α,β,β-TMN (1,3,7-TMN; aerobic: 84.11 ± 0.11%, anaerobic: 97.72 ± 0.01%) > α,α,β-TMN (1,4,6 + 1,3,5-TMN; aerobic: 74.23 ± 0.03%, anaerobic: 97.16 ± 0.02%) (Figure 5E). The biodegradation ratio of α,α-DMN (1,8-DMN: aerobic: 71.61 ± 0.10%, anaerobic: 92.35 ± 0.01%) was the lowest among all other configurations (Figure 5E). These results indicate that the biodegradability of alkylnaphthalenes with methyl substitutions at the β position was higher than that with α substituent.

Fluorenes are classified as one of the 16 priority-controlled PAHs (Zhan et al., 2018). The average biodegradation ratio of fluorene series under anaerobic condition (84.87 ± 0.03%) was higher than that under aerobic condition (64.48 ± 0.04%) (Figures 3, 5B). The presence of methyl groups decreased the biodegradation ratio, with fluorene (F, aerobic 82.81 ± 0.07%; anaerobic 93.38 ± 0.01%) showing a higher biodegradation ratio than methylated fluorenes (MFs, aerobic 63.30 ± 0.02%; anaerobic 84.32 ± 0.03%) (Figures 5F, 6).

Thiophenes are a class of sulfur-containing heterocyclic aromatic hydrocarbons (Mezcua et al., 2008; Li et al., 2014). The biodegradation ratios of dibenzothiophenes (DBTs) by CN32 were slightly higher under anaerobic (41.35 ± 0.02%) condition than under aerobic condition (40.44 ± 0.04%) (Figures 3, 5B). Under both conditions, the biodegradation ratio of DBTs decreased as the number of methyl groups increased (Figures 5F, 6).

Dibenzofurans are a class of oxygen-containing heterocyclic aromatic hydrocarbons (Li et al., 2018). CN32 showed a higher degradation ratio of dibenzofurans (DBFs) under anaerobic condition (95.37 ± 0.02%) compared to aerobic condition (83.11 ± 0.02%) (Figure 3).

Biphenyls (BPs) are bicyclic PAHs composed of two benzene rings. Similar to other PAHs, CN32 showed a higher degradation ratio of BPs under anaerobic condition than under aerobic condition (Figures 3, 5C). Similarly, BPs with a higher number of methyl substitutions showed a lower biodegradation ratio. For example, DMBPs (containing two methyl groups) exhibited higher degradation ratios compared to TMBPs (containing three methyl groups) (Figures 5G, 6).

The phenanthrene series contains three benzene rings (Wei et al., 2017), including phenanthrenes (Ps), methylphenanthrenes (MPs), and dimethylphenanthrenes (DMPs). Similarly to other PAHs, the degradation ratio of the phenanthrene series decreased as the number of methyl groups increased under both aerobic and anaerobic conditions (Figures 5D,H, 6), with P (aerobic 54.73 ± 0.06%; anaerobic 74.28 ± 0.05%) showing a higher biodegradation ratio than MPs (aerobic 34.75 ± 0.06%; anaerobic 42.60 ± 0.04%) and DMPs (aerobic 27.48 ± 0.06%; anaerobic 29.15 ± 0.04%) (Figures 5H, 6).

### 3.4 RT-qPCR results

**Figure 7:**
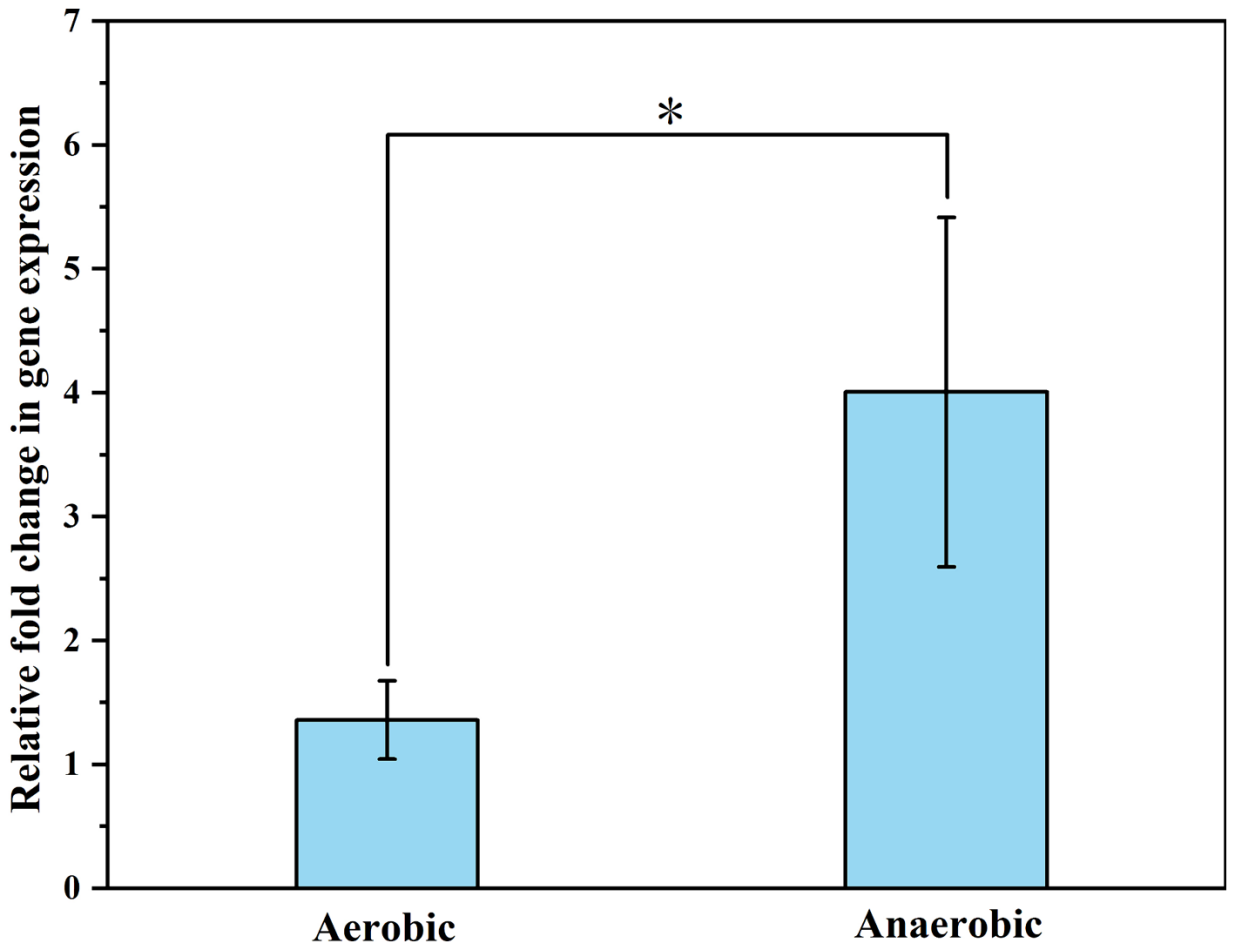
The expression of functional genes (ubiD) relevant to aromatic degradation by CN32 under aerobic and anaerobic conditions. 16S rRNA was used as a reference gene. *p* values from statistical analysis are shown with asterisks: <0.05, denoted by *.

The ratio of OD_260_ /OD_280_ of the extracted RNA was about 2.0 ± 0.1 (Table 2). Agarose gel electrophoresis showed clear bands of 23S and 16S subunits, indicating that the RNA was of high quality and did not degrade significantly. The samples exhibited Ct values below 35, and the melting curves for both the internal standard and the target genes showed a sole signal peak, providing clear evidence for a successful primer design. A nearly threefold increase in the relative expression levels of the ubiD gene was detected when CN32 was placed under anaerobic condition, as compared to aerobic conditions (Figure 7). The observed upregulation of the ubiD gene was consistent with the higher capacity of CN32 cells to break down aromatic compounds under anaerobic condition than under aerobic condition (Figure 3).

**Table 2:** The concentration and OD value of RNA extracted from *S. putrefaciens* CN32.

Condition	ID	Sample type	SW (nm)	SW Abs	260 Abs (10 mm)	280 Abs (10 mm)	260/280	Conc. (ng/μl)
Aerobic	1–1	RNA	260	2.555	2.555	1.239	2.062	102.2
	1–2	RNA	260	2.535	2.535	1.23	2.062	101.4
	1–3	RNA	260	2.548	2.548	1.255	2.03	101.92
Anaerobic	2–1	RNA	260	5.462	5.462	2.85	1.916	218.48
	2–2	RNA	260	5.193	5.193	2.668	1.946	207.72
	2–3	RNA	260	5.067	5.067	2.638	1.921	202.68

## 4 Discussion

### 4.1 Higher degradation of saturated hydrocarbons under aerobic condition than under anaerobic condition

The biodegradation ratio of *n*-alkanes by CN32 (71.26 ± 0.01%) under aerobic condition may be lower than those by obligately aerobic bacteria such as *Pseudomonas aeruginosa* XJ16 and *Bacillus cereus* XJ20 (98.2–98.8%) (Liu et al., 2020a). However, under anaerobic condition, the anaerobic biodegradation ratio of 25.16 ± 0.01% was much higher than ~7.5% achieved by an anaerobic community using Fe^3+^ as an electron acceptor over the same period (30 days) (Zhang et al., 2019). These unique capabilities expand the potential of CN32 for effective biodegradation of *n*-alkanes under a wide range of environmental conditions.

Bacterial utilization of *n*-alkanes is generally biased towards short carbon chain lengths under aerobic (Hasanuzzaman et al., 2007; Pasumarthi et al., 2013) and anaerobic condition (Rueter et al., 1994; Zhang et al., 2019). However, there are exceptions to this trend. For instance, *Acinetobacter lwoffii* XJ19 exhibited a higher capacity for biodegrading long-chain *n*-alkanes (> C_28_, 56.8–74.5%) compared to *n*C_15_ - *n*C_28_ (9.1–55.8%) under aerobic condition (Liu et al., 2020a). Similarly, Cheng et al. (2019) reported the preferential degradation of long-chain *n*-alkanes (>*n*C_19_) in heavy oil under methanogenic conditions. Therefore, the specific metabolic capability of bacteria plays a pivotal role in determining the efficiency of *n*-alkane degradation. CN32 demonstrated a remarkable ability of CN32 to degrade *n*-alkanes and alkylcyclohexanes with both short and long carbon chains under two conditions (Figure 3), which was first proposed. The presence of the alkB gene in the CN32 gene bank, as indicated by data from the NCBI database (see footnote 1), suggests its potential role in degrading *n*-alkanes with carbon numbers below 20 (Sierra-Garcia and de Oliveira, 2013). However, the genes responsible for biodegradation of long-chain *n*-alkanes, such as lutein-binding monooxygenase (almA; >*n*C_32_) (Wentzel et al., 2007) and long-chain alkane monooxygenase (ladA; *n*C_15_-*n*C_36_) (Feng et al., 2007), are absent in the CN32 genome. Therefore, the specific genes accountable for the aerobic biodegradation of *n*-alkanes with carbon numbers above 20 remain unidentified. Consequently, the molecular mechanisms governing the biodegradation of *n*-alkanes with varying carbon numbers cannot be elucidated. Further investigations are imperative to pinpoint and characterize the specific genes and enzymes involved in biodegradation of these longer-chain *n*-alkanes by CN32.

### 4.2 Higher degradation of aromatic hydrocarbons under anaerobic condition than under aerobic condition

Anaerobic degradation of PAHs is generally considered slower than aerobic degradation (Widdel et al., 2006). For example, when using O_2_ as an electron acceptor, a facultative bacterium completely degraded phenanthrene within 3 days, whereas when Fe(III) was used, it took 10 days to achieve complete degradation (Zhang et al., 2021). However, CN32 possesses a distinct ability to degrade PAHs more effectively under anaerobic condition than under aerobic condition (Figures 3, 5, 6). Grishchenkov et al. (2000) obtained similar conclusions when facultative nitrate-reducing bacteria were used to degrade PAHs with two rings (i.e., naphthalenes and fluorenes) under both anaerobic and aerobic conditions. However, the study reported a significantly lower degradation ratio for two-ring PAHs, with the highest level not exceeding 23%, which stands in stark contrast to the remarkable ~84% degradation ratio observed in the current study (i.e., naphehalenes, fluorenes, Dibenzofurans, and biphenyls). In fact, the anaerobic degradation ratio achieved by CN32 surpassed many of the published degradation ratios for PAHs by anaerobic microorganisms under nitrate-, ferric-, sulfate-reducing, and methanogenic conditions (Grishchenkov et al., 2000; Rockne et al., 2000; Chang et al., 2006; Zhang et al., 2019). This underscores the significant potential of CN32 for petroleum degradation in anaerobic environments. With the ability to degrade PAHs using either O_2_ or Fe(III) as electron acceptors, CN32 demonstrates a superior capability in utilizing different electron acceptors for PAH degradation.

The preferred degradation ability of CN32 under anaerobic condition can be attributed to the unique enzyme systems of this bacterium, because enzymes exhibit specificity for different compounds (Tani et al., 2001; Haritash and Kaushik, 2009; Sierra-Garcia and de Oliveira, 2013; Moreno and Rojo, 2017). Under anaerobic condition, the initial activation mechanisms of petroleum hydrocarbons include carboxylation (So et al., 2003), hydroxylation (Zhang et al., 2013), and fumarate addition (Wilkes et al., 2002). In the CN32 genome, there is a notable absence of conventional genes typically associated with fumarate addition (see footnote 1). However, our analysis reveals alternative mechanisms such as carboxylation, as evidenced by a significantly elevated expression of the ubiD gene (Figure 7). Indeed, a previous study by Zhang et al. (2021) demonstrated that carboxylation serves as the initial activation pathway for phenanthrene degradation under Fe(III)-reducing condition. Moreover, Atashgahi et al. (2018) and Koelschbach et al. (2019) reported the important role of ubiD-related carboxylases in the initial activation of benzene and naphthalene under nitrate- and sulfate-reducing conditions, respectively. These results suggest that carboxylation is a common activation pathway in anaerobic biodegradation of petroleum hydrocarbons. Furthermore, the CN32 genome harbors homologues of the benzoyl-CoA reductase (bamD) gene (Wischgoll et al., 2005) and the oxoenoyl-CoA hydrolase (bamA) gene (DiDonato Jr et al., 2010), suggesting their potential involvement in the reduction and cleavage of benzene rings during anaerobic degradation of PAHs. The synergy between the ubiD gene and the Bam system in CN32 may have enhanced its ability to effectively degrade PAHs under anaerobic conditions.

CN32 lacks genes encoding oxygenases for the hydroxylation and oxygenolytic ring cleavage of aromatic compounds under aerobic condition (Ghosal et al., 2016). Nevertheless, data from the KEGG database of degradation of aromatic compounds - *Shewanella putrefaciens* CN32 (https://www.kegg.jp/kegg-bin/show_pathway?spc01220) suggest possible involvement of dioxygenases in the degradation pathways of naphthalene, biphenyl, dibenzofuran, and phenanthrene under aerobic condition. Dioxygenases are recognized for their role in initiating the breakdown of aromatic rings (Bugg and Winfield, 1998). Remarkably, the complete genome of CN32 lacks typical genes associated with aerobic degradation of aromatic compounds, including those for naphthalene dioxygenase (NahAaAbAcAd) (Phale et al., 2019) and catechol 2,3-dioxygenase (C23O) (Fuentes et al., 2014). This leads us to conjecture that functionally analogous enzymes might be active within CN32, enabling it to metabolize aromatic compounds through alternate pathways. However, the specific genes and enzymes cannot be confirmed. Additional research is required to uncover the precise degradation genes and pathways utilized by CN32 for breaking down aromatic hydrocarbons under aerobic condition.

Besides the intrinsic biodegradability of CN32, the structure of the PAHs also exerts a significant influence on the degradation ratio. PAHs with three benzene rings (phenanthrenes) were less degraded compared to PAHs with two benzene rings under both conditions (Figures 3, 5, 6), and PAHs with more than three aromatic rings did not exhibit any significant biodegradation (Figure S2 in Supplementary material). Indeed, a previous study showed that PAHs with a higher number of aromatic rings exhibited increasing bio-resistance (George et al., 2002). The reason for this trend is that with the increasing number of benzene rings in PAHs, both the half-life times for PAH biodegradation and the resonance energy of their structures increase, which leads to their high thermodynamic stability and low bioavailability (Aronstein et al., 1991; Cerniglia, 1992; Morasch et al., 2011; Sharma et al., 2016). In addition, as the number of benzene rings and molecular weight of PAHs increase, the solubility of PAHs decreases, which leads to higher bio-resistance as well (Cerniglia, 1992; Dhar et al., 2020).

When the number of benzene rings is kept constant, PAHs were less degraded with the increased number of methyl groups (Figure 6). This matches what previous studies found, although they mainly focused on the specific compounds (Volkman et al., 1984; Chen et al., 2013) or specific environmental conditions (Liu et al., 2023b). In contrast, our study offers a comprehensive analysis of all series of dicyclic and tricyclic PHAs present in petroleum hydrocarbons, encompassing both aerobic and anaerobic conditions (Figure 6). The increased methyl substitutions may reduce the available sites for enzymatic activity, which in turn could diminish the biodegradation potential (Leblond et al., 2001). When the number of methyl groups remains constant, the position of these methyl becomes a crucial determinant of biodegradation ratios (Figures 5E-H). The presence of ortho substitutions introduces greater steric hindrance, consequently diminishing the biodegradation ratio (Liu et al., 2023b). Additionally, isomers featuring β-methyl substituents tend to undergo more rapid degradation compared to those with α-methyl substituents (Raymond et al., 1967; Volkman et al., 1984). Our study aligns with these findings, as illustrated in Figure 5E. It’s important to note that prior investigations exclusively focused on the aerobic degradation characteristics of alkylnaphthalenes (Raymond et al., 1967; Volkman et al., 1984; Liu et al., 2023b). In contrast, our study expands upon the understanding of alkylnaphthalenes degradation by examining its characteristics under anaerobic conditions.

### 4.3 Environmental implications

Facultative bacteria are commonly found and are prevalent in a wide range of environments (Table S1 in Supplementary material), because they adapt to diverse ecological niches (Tschech and Fuchs, 1987; Bae et al., 2006). These bacteria can thrive when oxygen is available and shift their metabolic pathways when oxygen becomes limited (Zhang et al., 2021). Thus, they contribute to the creation of biogeochemical gradients, such as redox gradients in sediments and water columns (Acosta-González and Marqués, 2016). Facultative bacteria are abundant in soil (Table S1 in Supplementary material), playing a crucial role in organic matter decomposition, element cycling, and the transformation of various compounds under changing oxygen conditions (McNally et al., 1998; Grishchenkov et al., 2000; Cason et al., 2019; Zuo et al., 2021; Sheng et al., 2021a; Dong et al., 2023a). In anaerobic environments, such as wetlands and sediments, facultative anaerobes can make up a substantial proportion of the microbial community (Bai et al., 2000; Zhu et al., 2011). In addition, ecosystems with fluctuating oxygen levels, like the water column in lakes and oceans, may have a significant presence of facultative bacteria (Haas et al., 2019). Some facultative bacteria are even found in extreme environments, such as hot springs, deep-sea hydrothermal vents, and acidic or alkaline environments (Raguénès et al., 1997; Pankratov et al., 2012; He et al., 2014; Mohr et al., 2018).

The versatility in adapting to changing conditions makes facultative bacteria valuable for breaking down complex molecules into simpler forms (McNally et al., 1998; Li et al., 2012; Zhang et al., 2021). As oxygen level decreases from the surface to subsurface environments, microorganisms can utilize iron-bearing minerals present in sediments as alternative electron acceptors for respiration (Favre et al., 2006; Han et al., 2020; Dong et al., 2022). Our results demonstrated that CN32 could utilize both O_2_ and Fe(III) as electron acceptors for oil degradation, expanding its potential for remediating oil-contaminated sites in aerobic and anaerobic zones. Similarly, Pathiraja et al. (2019) revealed that the facultative bacterium *Lysinibacillus* sp. NP05 exhibited a superior ability of polychlorinated biphenyls (PCBs) degradation under two-stage anaerobic-aerobic conditions compared to constant aerobic (using O_2_ as an electron acceptor) or anaerobic (using PCBs as an electron acceptor) conditions. Therefore, facultative bacteria like CN32 and NP05 could serve as effective alternatives to obligate bacteria for the degradation of organic pollutants under oscillating redox conditions. These findings highlight an advantage of facultative anaerobic bacteria in the continuous remediation of organic pollutants across diverse environments (Table S1 in Supplementary material).

We focused on the biodegradation of complex crude oil, in contrast to previous researches that primarily investigated the degradation of specific classes of petroleum hydrocarbons, such as phenanthrenes (Zhang et al., 2021), pyrene and benzo[*a*]pyrene (Liang et al., 2014; Yan et al., 2017), *n*-alkanes, naphthalenes, and fluorenes (Grishchenkov et al., 2000). Thus, this study provides valuable insights for developing targeted pollution remediation strategies by uncovering specific degradation capabilities of CN32 under different conditions. This bacterium can aerobically degrade *n*-alkane pollutants at the surface where oxygen is abundant. In the subsurface, where oxygen is limited but dissolved Fe(III), iron-bearing minerals, or iron (oxyhydr)oxides may be abundant, this bacterium switches to anaerobic degradation of aromatics. Thus, CN32 is a valuable microorganism for remediating oil pollutants in various environmental settings, including soil, groundwater, seafloor, and transitional zones between terrestrial and marine. The dual capabilities of CN32 to target both saturated and aromatic hydrocarbons under aerobic and anaerobic conditions highlight its versatility as a bioremediation agent for diverse hydrocarbon pollutants. Furthermore, it is also noteworthy that facultative bacteria have an advantage in pollutant degradation due to their ability to generate hydroxyl radicals through microbial-driven Fenton reaction, utilizing H_2_O_2_ generated during aerobic respiration or Fe(II) generated during anaerobic Fe(III) reduction (Sekar and DiChristina, 2014; Han et al., 2020, 2022). Hydroxyl radicals possess strong oxidizing capabilities and can chemically oxidize petroleum hydrocarbons (Tsai and Kao, 2009; Cheng et al., 2016). This approach not only offers a more cost-effective and environmentally friendly alternative to chemical Fenton reactions (Klečka and Gonsior, 1986; Stefan and Bolton, 1998), but also achieves faster degradation rates compared to traditional bioremediation methods (Shen et al., 2008; Kim et al., 2009). Therefore, the application potential of CN32 in this field can be further explored.

## 5 Conclusion

In this study, degradation of crude oil by a facultative anaerobic iron-reducing bacterium *S. putrefaciens* CN32 was investigated under aerobic and anaerobic conditions using O_2_ and Fe(III) as electron acceptors. The results unveiled intriguing patterns, revealing that saturates were more susceptible to biodegradation under aerobic conditions, exhibiting a remarkable average biodegradation ratio of 65.65 ± 0.01%, higher than that of aromatics at 43.86 ± 0.03%. Notably, the degradation of saturated hydrocarbons displayed a complex pattern, with the biodegradation ratio initially decreasing and then increasing as the carbon chain length increased. Conversely, aromatics were more susceptible to biodegradation under anaerobic condition than under aerobic condition, due to the higher ubiD gene expression level under anaerobic condition. Furthermore, under anaerobic condition, CN32 demonstrated a preference for degrading aromatics (53.94 ± 0.02%) over saturates (23.36 ± 0.01%). Additionally, as the number of aromatic rings and methyl groups in PAHs increased, aromatic hydrocarbons became more resistant to biodegradation. These profound findings not only illuminate the intricate degradation pathways of crude oil by *S. putrefaciens* CN32, but also offer invaluable insights for the selection of microorganisms with specific fingerprint for targeted organic pollution remediation in both aerobic and anaerobic environments.

## Data availability statement

The original contributions presented in the study are included in the article/Supplementary material, further inquiries can be directed to the corresponding author.

## Author contributions

YaL: Data curation, Formal analysis, Investigation, Methodology, Visualization, Writing – original draft. YuL: Conceptualization, Data curation, Formal analysis, Funding acquisition, Methodology, Visualization, Writing – review & editing. DG: Visualization, Writing – review & editing. HD: Resources, Supervision, Writing – review & editing, Funding acquisition.

## Footnotes

^1^ https://www.ncbi.nlm.nih.gov/
