# Supplementary material for: Differential degradation of petroleum hydrocarbons by Shewanella putrefaciens under aerobic and anaerobic conditions
Source: Front Microbiol. 2024 Apr 10;15:1389954. doi: 10.3389/fmicb.2024.1389954 (PMC11040095; doi:10.3389/fmicb.2024.1389954)
Supplement: Supplementary file 1 [file Table_1.doc]

Supporting Information for

**Differential degradation of petroleum hydrocarbons by *Shewanella putrefaciens* under aerobic and anaerobic conditions**

Yang Li ^a,b^,^†^ Yuan Liu ^a,b^,^†^ Dongyi Guo ^a^, Hailiang Dong ^a,b^,*

^a^ Center for Geomicrobiology and Biogeochemistry Research, State Key Laboratory of Biogeology and Environmental Geology, China University of Geosciences, Beijing 100083, China

^b^ School of Earth Sciences & Resources, China University of Geosciences, Beijing 100083, China

†: co-lead authors

*Corresponding author at: Center for Geomicrobiology and Biogeochemistry Research

State Key Laboratory of Biology and Environmental Geology, China University of Geosciences, Beijing 100083, China

*E-mail address*: [dongh@cugb.edu.cn](mailto:dongh@cugb.edu.cn) (H. Dong)

10 Pages Total

1 Text

1 Tables

2 Figures

**Text S1.**

GC-MS analyses of saturated and aromatic hydrocarbons were conducted using a Thermo Finnigan Trace-DSQ mass spectrometer coupled to an HP 6890 GC equipped with an HP-5MS column (30 m × 0.25 mm ID) with a 0.25-μm coating. Helium was used as the carrier gas. The GC oven temperature was initially set at 50 °C, after which it was increased to 120 °C at a rate of 20 °C/min, 250 °C at a rate of 4 °C/min, and 310 °C at a rate of 3 °C/min and maintained at this temperature for 30 mins. The mass spectrometer was operated in the full-scan, electron impact mode with an electron energy of 70 eV .

**Table S1** Facultative bacteria from diverse environments participate in redox coupling through the utilization of varying electron acceptors and organic compounds

| Origin of samples | Strains | Electron donors | | Electron acceptors | | Biodegradation | References |
| --- | --- | --- | --- | --- | --- | --- | --- |
| Sediments | *Pseudomonas* sp. JP1 | **benzo[a]-pyrene** | | aerobic: O_2_ | | 57% |  |
|  |  |  |  | anaerobic | nitrate | ~30% |  |
|  |  |  |  |  | sulfite | ~45% |  |
|  |  |  |  |  | **Fe(III)** | **~33%** |  |
|  |  |  |  |  | Mn (IV) | ~26% |  |
|  |  | **fluoranthene** | | aerobic: O_2_ | | 45% |  |
|  |  |  |  | anaerobic: nitrate | | ~47% |  |
|  |  | **phenanthrene** | | aerobic: O_2_ | | 5% |  |
|  |  |  |  | anaerobic: nitrate | | ~5% |  |
|  | *Hydrogenophaga* sp. PYR1 | **pyrene** | | aerobic: O_2_ | | 94% |  |
|  |  |  |  | anaerobic: **Fe(III)** | | **~21%** |  |
|  |  | **benzo[a]-pyrene** | | aerobic: O_2_ | | No degradation |  |
|  |  |  |  | anaerobic: **Fe(III)** | | **~24%** |  |
|  | *Bacillus* sp. GZB | bisphenol A | | aerobic: O_2_; | | 100% |  |
|  |  |  |  | anaerobic: **Fe(III)** | | **100%** |  |
|  | *Shewanella saccharophilia* strain GC-29 | glucose | | aerobic: O_2_ | | completely oxidized glucose to CO_2_ with no production of acetate |  |
|  |  |  |  | anaerobic: **Fe(III)** | | **the accumulation of acetate** |  |
|  | *Rhodoferax ferrireducens* sp. T118^T^ | acetate, lactate, malate, propionate, pyruvate, succinate, and benzoate | | anaerobic: **Fe(III)** | |  |  |
|  | *Paracoccus denitrificans* strain M-1 | **pyrene** | Low N amended (0 mM) | anaerobic: nitrate and nitrite | | 75% |  |
|  |  |  | High N amended (5 mM) |  |  | 47% |  |
|  |  |  | Higher N amended (8 mM) |  |  | 35% |  |
|  | strain DEA 4 | diethanolamine | | aerobic: O_2_ | | 70% |  |
|  |  |  |  | anaerobic: nitrate | | 70% |  |
|  | *Vibrio pelagius* NAP-4 | **naphthalene** | | anaerobic: nitrate | | 70% |  |
|  | *Pseudomonas stutzeri* NAP-3-1 |  |  |  |  | 90% |  |
|  | Facultative anaerobic flora | Biological Oxygen Demand (BOD_5_) | | aerobic: O_2_ | | >90% |  |
| Soil and bay sediment | *Pseudomonas stutzeri* SAG-R | **phenanthrene** | | aerobic: O_2_ | | 100% within 12 h |  |
|  |  |  |  | anaerobic: nitrate | | 100% within 12 h |  |
|  |  | **pyrene** | | aerobic: O_2_ | | 100% within 24 h |  |
|  |  |  |  | anaerobic: nitrate | | 100% within 24 h |  |
|  | *Pseudomonas putida* KBM-1 |  |  | aerobic: O_2_ | | 100% within 60 h |  |
|  |  |  |  | anaerobic: nitrate | | 100% within 72 h |  |
|  |  | **phenanthrene** | | aerobic: O_2_ | | 100% within 12 h |  |
|  |  |  |  | anaerobic: nitrate | | 100% within 44 h |  |
|  |  | **anthracene** | | aerobic: O_2_ | | 100% within 12 h |  |
|  |  |  |  | anaerobic: nitrate | | 100% within 60 h |  |
|  | *Pseudomonas fluorescens* W-2 |  |  | aerobic: O_2_ | | 100% over 84 h |  |
|  |  |  |  | anaerobic: nitrate | | 100% within 40 h |  |
| River sediments, sludge, and soil | *Pseudomonads* sp. S100 and K172 | phenol, cresols, aromatic acids | | aerobic: O_2_ | |  |  |
|  |  |  |  | anaerobic: nitrate or nitrite | |  |  |
| Soil and river sediments | *Achromobacter* sp. NP03, *Ochrobactrum* sp. NP04, *Lysinibacillus* sp. NP05 and *Pseudomonas* sp. NP06 | aerobic: polychlorinated biphenyls (PCBs) | | aerobic: O_2_ | | ~3.3%-8.3% |  |
|  |  | anaerobic: H_2_ | | anaerobic: PCBs | | ~5.7%-17.3% |  |
|  |  | two stage anaerobic-aerobic condition | | | | ~12.3%-30.5% |  |
|  | bacterial consortium  (*Achromobacter* sp. NP03, *Ochrobactrum* sp. NP04 and *Lysinibacillus* sp. NP05) |  |  |  |  | 24.44 ± 2.46%; |  |
|  |  | alternating anaerobic-aerobic condition | | | | 49.2 ± 2.5% |  |
|  | *Chromobacterium alkanivorans* sp. IITR-71^T^ | 1-chloropropane | | aerobic: O_2_ | | 83.19% |  |
|  |  | 1-chlorobutane | |  |  | 79.64% |  |
|  |  | 1,2-dichlorethane | |  |  | 37.09% |  |
| Soil | *Microbacterium* sp*.* | **benzo[a]pyrene** | | anaerobic: nitrate | | 84.3% |  |
|  | *Cellulosimicrobium cellulans* CWS2 | **benzo[a]pyrene** | | anaerobic: nitrate | | 78.8% |  |
|  | *Pseudomonas* sp. BS2201, BS2203 and *Brevibacillus* sp. BS2202 | **crude oil (*n*-alkanes)** | | aerobic: O_2_ | | 90%-95% |  |
|  |  |  |  | anaerobic: nitrate | | 20%-25% |  |
|  |  | **crude oil (aromatics)** | | aerobic: O_2_ | | No degradation |  |
|  |  |  |  | anaerobic: nitrate | | <23% |  |
|  | *Pseudomonas* PN-1 | vanillate | | aerobic: O_2_ | | 71% |  |
|  |  |  |  | anaerobic: nitrate | | 73.5% |  |
|  |  | vanillin | | aerobic: O_2_ | | 26.5% |  |
|  |  |  |  | anaerobic: nitrate | | 100% |  |
|  |  | vanillyl alcohol | | aerobic: O_2_ | | 18.5% |  |
|  |  |  |  | anaerobic: nitrate | | 60.5% |  |
|  | soil enrichments  (*Pseudomonas* spp. and *Citrobacter* spp.) | diesel | | aerobic: O_2_ | | Star Diamonds Soil Enrichment: better aerobic degradation |  |
|  |  |  |  | anaerobic: nitrate, sulfite | | Free State Groundworks Soil Enrichment: better anaerobic degradation |  |
| Soil, sludge | Strain Phe F2 | **phenanthrene** | | aerobic: O_2_ | | 100% within 3 days |  |
|  |  |  |  | anaerobic: **Fe(III)** | | **100%** within 10 days |  |
| Sludge | *Aquabacterium sp.* CZ3 | phenol | | anaerobic: nitrate | | 100% |  |
|  | *Citrobacter* AzoR-1*, Acinetobacter* AzoR-3*, Pseudomonas* AzoR-9*, and Bacillus* AzoR-6 | H_2_ and formate, lactate, and pyruvate | | amaranth | | 100% |  |
|  | microbial consortium  (*Chromobacterium*) | 1,2-Dibromoethane | | aerobic: O_2_ | | >61% |  |
|  |  |  |  | anaerobic: fermentation | |  |  |
|  | *Staphylococcus arlettae* strain VN-11 | azo dyes | | aerobic: O_2_ | | >97% |  |
|  | *Enterobacter* DG-6 | 3-methoxy-4-hydroxycinnamate (ferulate) | | aerobic: O_2_ | | 99.4% |  |
|  |  |  |  | anaerobic: fermentation | |  |  |
| Contaminated groundwater | *Propionicicella superfundia* strain BL-10^T^ | glucose | | anaerobic: fermentation | |  |  |
| Oilfield | *Rhodococcus ruber* Z25 | **Crude oil** | | aerobic: O_2_ | | aerobic degradation is more significant than anaerobic degradation. |  |
|  |  |  |  | anaerobic: nitrate | |  |  |
|  | *Chelatococcus daeguensis* HB-4 |  |  | aerobic: O_2_ | | 48.9% |  |
|  |  |  |  | anaerobic: fermentation | | 27.2% |  |
|  | *Klebsiella oxytoca* strain BSC5 |  |  | aerobic: O_2_ | | *n*-alkanes (75% and 98% with Tween 80) |  |

Note: in bold, petroleum hydrocarbons act as electron donors, or Fe (III) as electron acceptors.

**Figure S1**. Concentration (μg/g) changes of steranes (A) and hopanes (B) due to aerobic (A-30) and anaerobic (An-30) biodegradation by CN32 for 30 days.

**Figure S2**. Concentration (μg/g) changes of PAHs with more than three rings due to aerobic (A-30) and anaerobic (An-30) biodegradation by CN32 for 30 days.

**Reference**

Bae, H.-S., Moe, W.M., Yan, J., Tiago, I., da Costa, M.S., and Rainey, F.A. (2006). Propionicicella superfundia gen. nov., sp. nov., a chlorosolvent-tolerant propionate-forming, facultative anaerobic bacterium isolated from contaminated groundwater. *Systematic and Applied Microbiology* 29**,** 404-413. doi: 10.1016/j.syapm.2005.11.004.

Bajaj, A., Kumar, A., Yadav, S., Kaur, G., Bala, M., Singh, N.K., et al. (2016). Isolation and characterization of a novel Gram-negative bacterium Chromobacterium alkanivorans sp. nov., strain IITR-71T degrading halogenated alkanes. *International Journal of Systematic and Evolutionary Microbiology* 66**,** 5228-5235. doi: 10.1099/ijsem.0.001500.

Cason, E.D., Vermeulen, J.-G., Müller, W.J., van Heerden, E., and Valverde, A. (2019). Aerobic and anaerobic enrichment cultures highlight the pivotal role of facultative anaerobes in soil hydrocarbon degradation. *Journal of Environmental Science & Health Part A Toxic/hazardous Substances & Environmental Engineering* 54(5)**,** 408-415. doi: 10.1080/10934529.2018.1558902.

Chamkha, M., Trabelsi, Y., and Sayade, S.M.S. (2011). Isolation and characterization of Klebsiella oxytoca strain degrading crude oil from a Tunisian off-shore oil field *Journal of Basic Microbiology* 51**,** 580-589. doi: 10.1002/jobm.201100073.

Coates, J.D., Councell, T., Ellis, D.J., and Lovley, D.R. (1998). Carbohydrate oxidation coupled to Fe(III) reduction, a novel form of anaerobic metabolism. *Anaerobe* 4**,** 277-282. doi: 10.1006/anae.1998.0172.

Elisangela, F., Andrea, Z., Fabio, D.G., de Menezes Cristiano, R., Regina, D.L., and Artur, C.P. (2009). Biodegradation of textile azo dyes by a facultative Staphylococcus arlettae strain VN-11 using a sequential microaerophilic/aerobic process. *International Biodeterioration & Biodegradation* 63**,** 280-288. doi: 10.1016/j.ibiod.2008.10.003.

Finneran, K.T., Johnsen, C.V., and Lovley, D.R. (2003). Rhodoferax ferrireducens sp. nov., a psychrotolerant, facultatively anaerobic bacterium that oxidizes acetate with the reduction of Fe(III). *International Journal of Systematic and Evolutionary Microbiology* 53**,** 669-673. doi: 10.1099/ijs.0.02298-0.

Grbić-Galić, D. (1985). Fermentative and Oxidative Transformation of Ferulate by a Facultatively Anaerobic Bacterium Isolated from Sewage Sludge. *Applied and Environmental Microbiology* 50(4)**,** 1052-1057. doi: 10.1128/aem.50.4.1052-1057.1985.

Grishchenkov, V., Townsend, R., McDonald, T., Autenrieth, R., Bonner, J., and Boronin, A. (2000). Degradation of petroleum hydrocarbons by facultative anaerobic bacteria under aerobic and anaerobic conditions. *Process Biochemistry* 35(9)**,** 889-896. doi: 10.1016/s0032-9592(99)00145-4.

Hong, Y., Guo, J., and Sun, G. (2008). Characteristics and phylogenetic analysis of the facultative anaerobic dissimilatory azoreducing bacteria from activated sludge. *International Biodeterioration & Biodegradation* 61**,** 313-318. doi: 10.1016/j.ibiod.2007.10.007.

Ke, C.-Y., Lu, G.-M., Wei, Y.-L., Sun, W.-J., Hui, J.-F., Zheng, X.-Y., et al. (2019). Biodegradation of crude oil by Chelatococcus daeguensis HB-4 and its potential for microbial enhanced oil recovery (MEOR) in heavy oil reservoirs. *Bioresource Technology* 287**,** 121442. doi: 10.1016/j.biortech.2019.121442.

Knapp, J.S., Jenkey, N.D., and Townsley, C.C. (1996). The anaerobic biodegradation of diethanolamine by a nitrate reducing bacterium *Biodegradation* 7**,** 183-189. doi: 10.1007/BF00058178.

Li, G., Zu, L., Wong, P.-K., Hui, X., Lu, Y., Xiong, J., et al. (2012). Biodegradation and detoxification of bisphenol A with one newly-isolated strain Bacillus sp. GZB: Kinetics, mechanism and estrogenic transition. *Bioresource Technology* 114**,** 224-230. doi: 10.1016/j.biortech.2012.03.067.

Liang, L., Song, X., Kong, J., Shen, C., Huang, T., and Hu, Z. (2014). Anaerobic biodegradation of high-molecular-weight polycyclic aromatic hydrocarbons by a facultative anaerobe Pseudomonas sp. JP1. *Biodegradation* 25**,** 825–833. doi: 10.1007/s10532-014-9702-5.

McNally, D.L., Mihelcic, J.R., and Lueking, D.R. (1998). Biodegradation of three-and four-ring polycyclic aromatic hydrocarbons under aerobic and denitrifying conditions. *Environmental Science & Technology* 32(17)**,** 2633–2639. doi: 10.1021/es980006c.

Pathiraja, G., Egodawatta, P., Goonetilleke, A., and Te'o, V.S.J. (2019a). Effective degradation of polychlorinated biphenyls by a facultative anaerobic bacterial consortium using alternating anaerobic aerobic treatments. *Science of the Total Environment* 659(1)**,** 507-514. doi: 10.1016/j.scitotenv.2018.12.385.

Pathiraja, G., Egodawatta, P., Goonetilleke, A., and Te'o, V.S.J. (2019b). Solubilization and degradation of polychlorinated biphenyls (PCBs) by naturally occurring facultative anaerobic bacteria. *Science of the Total Environment* 651(1)**,** 2197-2207. doi: 10.1016/j.scitotenv.2018.10.127.

Qin, W., Fan, F., Zhu, Y., Huang, X., Ding, A., Liu, X., et al. (2018). Anaerobic biodegradation of benzo(a)pyrene by a novel Cellulosimicrobium cellulans CWS2 isolated from polycyclic aromatic hydrocarbon-contaminated soil. *Brazilian Journal of Microbiology* 49**,** 258-268. doi: 10.1016/j.bjm.2017.04.014.

Qin, W., Zhu, Y., Fan, F., Wang, Y., Liu, X., Ding, A., et al. (2017). Biodegradation of benzo (a) pyrene by Microbacterium sp. strain under denitrification: degradation pathway and effects of limiting electron acceptors or carbon source. *Biochemical Engineering Journal* 121**,** 131-138. doi: 10.1016/j.bej.2017.02.001.

Rockne, K.J., Chee-Sanford, J.C., Sanford, R.A., Hedlund, B.P., Staley, J.T., and Strand, S.E. (2000). Anaerobic naphthalene degradation by microbial pure cultures under nitrate-reducing conditions. *Applied and Environmental Microbiology* 66(4)**,** 1595-1601. doi: 10.1128/AEM.66.4.1595-1601.2000.

Taylor, B.F. (1983). Aerobic and Anaerobic Catabolism of Vanillic Acid and Some Other Methoxy-Aromatic Compounds by Pseudomonas sp. Strain PN-1. *Applied and Environmental Microbiology* 46(4)**,** 1286-1292. doi: 10.1128/aem.46.6.1286-1292.1983.

Tschech, A., and Fuchs, G. (1987). Anaerobic degradation of phenol by pure cultures of newly isolated denitrifying pseudomonads. *Archives of Microbiology* 148**,** 213-217. doi: 10.1007/BF00414814.

Wang, Q., Yang, M., Song, X., Tang, S., and Yu, L. (2019). Aerobic and Anaerobic Biodegradation of 1,2-Dibromoethane by a Microbial Consortium under Simulated Groundwater Conditions. *International Journal of Environmental Research and Public Health* 16**,** 3775. doi: 10.3390/ijerph16193775.

Yan, Z., Zhang, Y., Wu, H., Yang, M., Zhang, H., Hao, Z., et al. (2017). Isolation and characterization of a bacterial strain Hydrogenophaga sp. PYR1 for anaerobic pyrene and benzo[a]pyrene biodegradation. *RSC advances* 7(74)**,** 46690-46698. doi: 10.1039/c7ra09274a.

Yang, X., Ye, J., Lyu, L., Wu, Q., and Zhang, R. (2013). Anaerobic biodegradation of pyrene by Paracoccus denitrificans under various nitrate/nitrite-reducing conditions. *Water, Air, & Soil Pollution* 224**,** 1578. doi: 10.1007/s11270-013-1578-1.

Zhang, R., Wang, X., Ali, A., Su, J., Wang, Z., Li, J., et al. (2022). Single-step removal of calcium, fluoride, and phenol from contaminated water by Aquabacterium sp. CZ3 via facultative anaerobic microbially induced calcium precipitation: Kinetics, mechanism, and characterization *Bioresource Technology* 361**,** 127707. doi: 10.1016/j.biortech.2022.127707.

Zhang, Z., Guo, H., Sun, J., Gong, X., Wang, C., and Wang, H. (2021). Exploration of the biotransformation processes in the biodegradation of phenanthrene by a facultative anaerobe, strain PheF2, with Fe (III) or O2 as an electron acceptor. *Science of The Total Environment* 750**,** 142245. doi: 10.1016/j.scitotenv.2020.142245.

Zheng, C., Yu, L., Huang, L., Xiu, J., and Huang, Z. (2012). Investigation of a hydrocarbon-degrading strain, Rhodococcus ruber Z25, for the potential of microbial enhanced oil recovery. *Journal of Petroleum Science and Engineering* 81**,** 49-56. doi: 10.1016/j.petrol.2011.12.019.

Zhu, Q., Ye, C., Li, C., Ke, L., and Zhao, X. (2011). Isolation and Degradation Effect of Facultative Anaerobic Flora with High Efficiency Organic Matter Biodegradation from Near-Shore Wetland in Taihu Lake. *Research of Environmental Sciences* 24(10)**,** 1129-1135. doi: 10.13198/j.res.2011.10.51.zhuqf.007.
