# Supplementary figures and images for: Differential degradation of petroleum hydrocarbons by Shewanella putrefaciens under aerobic and anaerobic conditions
Source: Front Microbiol. 2024 Apr 10;15:1389954. doi: 10.3389/fmicb.2024.1389954 (PMC11040095; doi:10.3389/fmicb.2024.1389954)

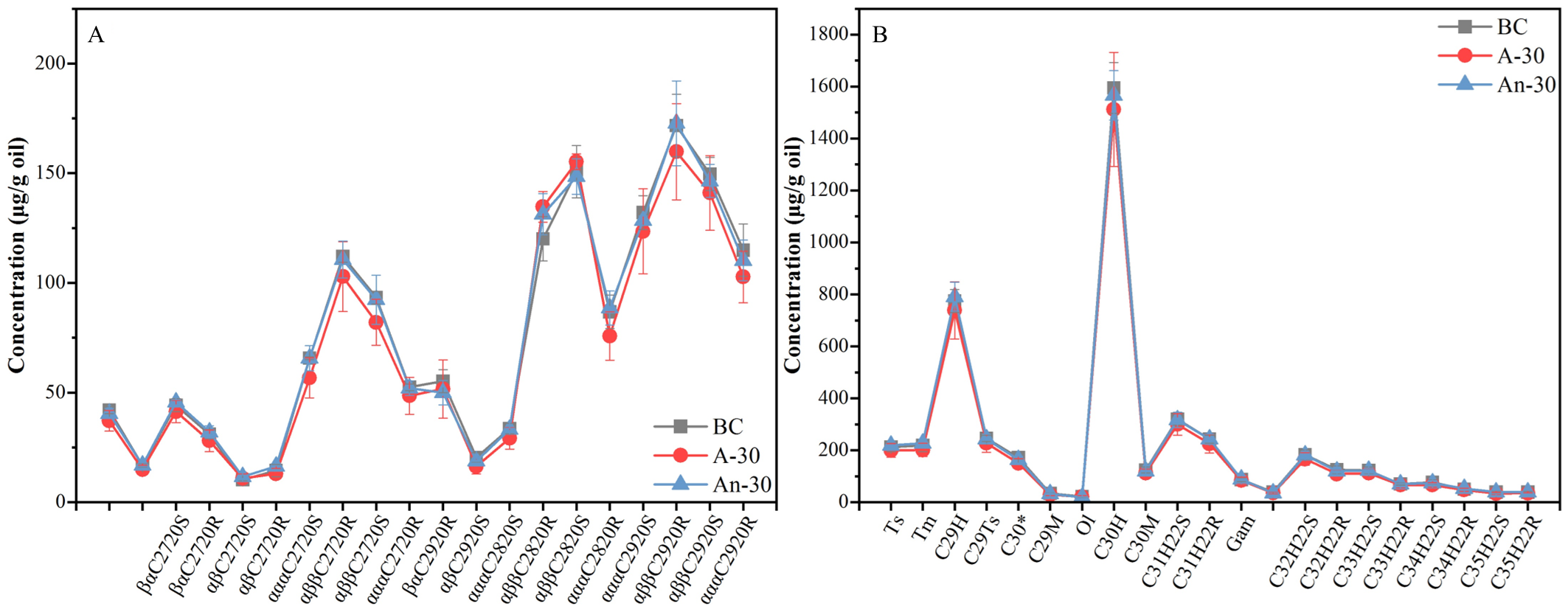

Supplement: Supplementary file 2 [file Image_1.TIF]

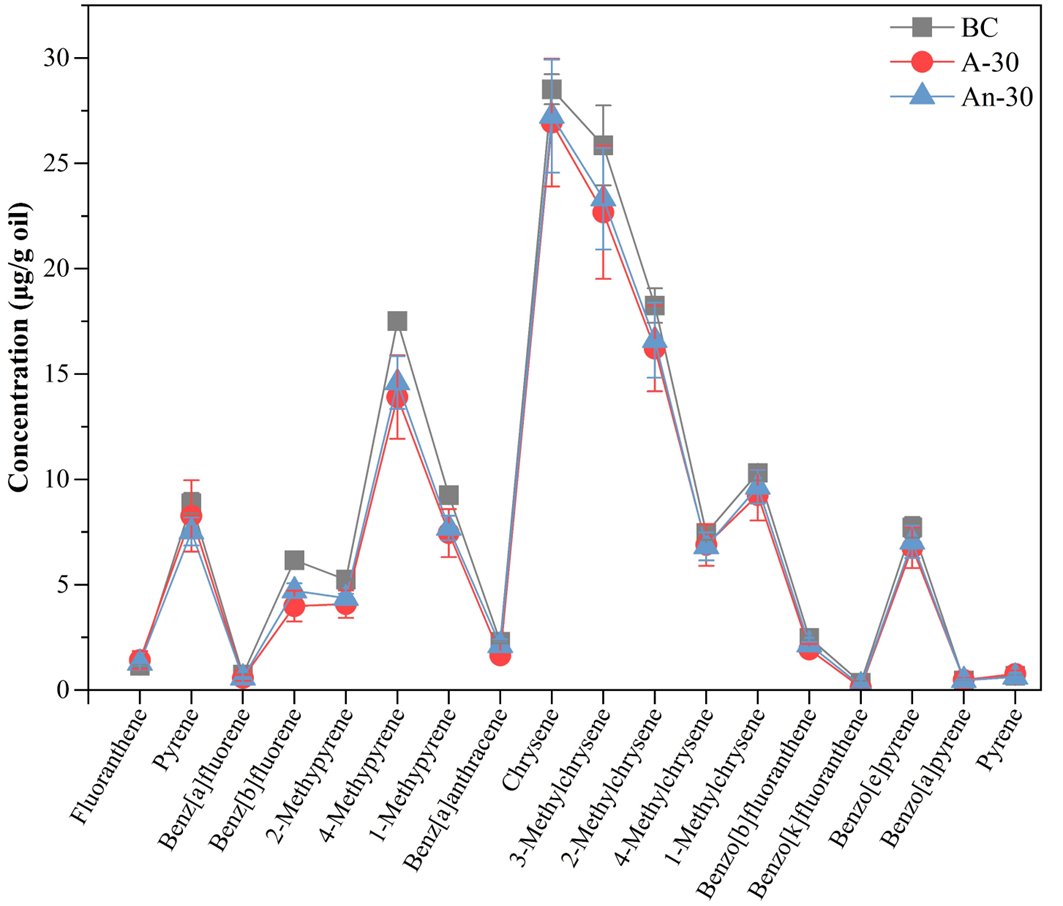

Supplement: Supplementary file 3 [file Image_2.TIF]
